# Genomic Features of Solid Tumor Patients Harboring *ALK/ROS1/NTRK* Gene Fusions

**DOI:** 10.3389/fonc.2022.813158

**Published:** 2022-06-16

**Authors:** Yinghuan Dai, Ping Liu, Wenlong He, Lizhen Yang, Yang Ni, Xuejiao Ma, Furong Du, Chao Song, Yang Liu, Yi Sun

**Affiliations:** ^1^ Department of Pathology, The Second Xiangya Hospital, Central South University, Changsha, China; ^2^ Department of Oncology, The Second Xiangya Hospital, Central South University, Changsha, China; ^3^ Department of Respiratory and Critical Care Medicine, The Second Xiangya Hospital, Central South University, Changsha, China; ^4^ State Key Laboratory of Translational Medicine and Innovative Drug Development, Jiangsu Simcere Diagnostics Co., Ltd., Nanjing, China; ^5^ Department of Medicine, Nanjing Simcere Medical Laboratory Science Co., Ltd., Nanjing, China; ^6^ Department of Thoracic Surgery, The First Affiliated Hospital of China Medical University, Shenyang, China

**Keywords:** *ALK*, *ROS1*, *NTRK*, gene fusion, next generation sequencing, mutational signature, copy number variants, programmed death ligand 1

## Abstract

The fusions of receptor tyrosine kinase (RTK) involving anaplastic lymphoma kinase (*ALK*), c-ros oncogene 1 (*ROS1*), and neurotrophic receptor tyrosine kinase (*NTRK*) represent the potential targets of therapeutic intervention for various types of solid tumors. Here, the genomic features of 180 Chinese solid tumor patients with *ALK*, *ROS1*, and *NTRK* fusions by next generation sequencing (NGS) were comprehensively characterized, and the data from 121 patients in Memorial Sloan Kettering Cancer Center (MSKCC) database were used to compare. We found that *ALK*, *ROS1*, and *NTRK* fusions were more common in younger female patients (*p*<0.001) and showed a higher expression of programmed death ligand 1 (PD-L1). The gene-intergenic fusion and the fusion with rare formation directions accounted for a certain proportion in all samples and 62 novel fusions were discovered. Alterations in *TP53* and *MUC16* were common in patients with RTK fusions. The mutational signatures of patients were mainly distributed in COSMIC signature 1, 2, 3, 15 and 30, while had a higher frequency in copy number variations (CNVs) of individual genes, such as *IL-7R*. In the MSKCC cohort, patients with fusions and CNVs showed shorter overall survival than those with only fusions. Furthermore, the differentially mutated genes between fusion-positive and -negative patients mainly concentrated on MAPK signaling and FOXO signaling pathways. These results may provide genomic information for the personalized clinical management of solid tumor patients with *ALK*, *ROS1*, and *NTRK* fusions in the era of precision medicine.

## Introduction

Chromosomal inversions, deletions or translocations leading to the constitutive activation of receptor tyrosine kinase (RTK) drive tumorigenesis across different malignancies (1, 2). The prevalence of RTK fusions involving anaplastic lymphoma kinase (*ALK*), c-ros oncogene 1 (*ROS1*), and neurotrophic receptor tyrosine kinase (*NTRK*) ranges from 0.3% to 5% in solid tumors and tyrosine kinase inhibitors (TKIs) are the standard treatment modality for the first-line setting of patients with advanced cancer harboring such fusions (3–5). Currently, multiple *ALK* fusion partners have been identified, of which echinoderm microtubule-associated protein-like 4 (*EML4*) is the most frequent, with nine variants occurring in nearly 80% of all the *ALK* fusion cases of non-small cell lung cancer (NSCLC) (6–8). Meanwhile, many different 5´ gene partners have been identified in the fusion with 3´ regions of *ROS1*. These fusions are discovered in adult glioblastoma, paediatric glioma, NSCLC, and inflammatory myofibroblastic tumor (IMTs) (4, 9). Additionally, approximately 80 *NTRK* fusion partners have also been described (10, 11). Although the frequency of *NTRK* fusions is low, they are ubiquitous in rare cancer types, such as mammary analog secretory carcinoma and infantile fibrosarcoma (12–15).

In recent years, a lot of clinical trials on treatments targeting specific molecular mechanisms like *ALK*, *ROS1*, and *NTRK* fusions have been conducted. Small molecule inhibitors for *ALK*, *ROS1*, and *NTRK* fusions, such as crizotinib, brigatinib, lorlatinib, entrectinib and larotrectinib, have been approved by the US Food and Drug Administration (FDA) for different cancer types (16–20). Despite the potential benefit from identifying these fusions, it remains unclear whether the tumors with *ALK*, *ROS1*, and *NTRK* fusions represent a distinct, although rare, disease subtype that should be detected early for targeted therapy. Herein, a comprehensive study was carried out to characterize the molecular and clinicopathological characteristics, and prognosis of solid tumor patients with *ALK*, *ROS1*, and *NTRK* fusions.

## Materials and Methods

### Sample Collection

In this study, the sequencing data of 7,537 solid tumor samples from the database of Simcere Diagnostics, Co. Ltd. (Nanjing, China) for genomic profiling between June 2019 and November 2020 were retrospectively analyzed, including lung cancer (n=3001), liver cancer (n=762), soft tissue sarcoma (n=281), bile duct carcinoma (n=232), esophageal cancer (n=155), breast cancer (n=154), melanoma (n=125), gallbladder carcinoma (n=121), bone tumor (n=59) and other unspecified tumors (n=2647) (**Table 1**). All patients signed written informed consents. The formalin fixed, paraffin-embedded (FFPE) tissue samples were selected for analysis, and peripheral blood samples were collected as the control. Here, 121 *ALK*, *ROS1*, and *NTRK* fusion-positive cancer patients from the MSK-IMPACT Clinical Sequencing Cohort (MSKCC, Nat Med 2017), which was composed of 10,945 samples, were used as the compared cohort.

**Table 1:** Patients’ characteristics according to the presence or absence of *ALK*, *ROS1*, and *NTRK* fusions.

Characteristics	ALK/ROS1/NTRK fusion negative (n=7357)	ALK/ROS1/NTRK fusion positive (n=180)	*p*	ALK fusion positive (n=103)	*p*	ROS1 fusion positive (n=40)	*p*	NTRK fusion positive (n=37)	*p*
Age, years*			0.0002		0.0166		0.0091		0.1514
Median	61	55		57		53.5		56.5	
Range	0-107	3-83		15-82		32-77		3-83	
Gender**			0.0137		0.1906		0.0006		0.8671
Female	2949	89		48		27		14	
Male	4404	91		55		13		23	
Pathology			<0.0001		<0.0001		0.0005		<0.0001
Bile duct carcinoma	229	3		0		1		2	
Bone tumor	56	3		1		0		2	
Breast cancer	152	2		0		0		2	
Esophageal cancer	153	2		1		1		0	
Gallbladder carcinoma	118	3		0		0		3	
Lung cancer	2865	136		93		33		10	
Liver cancer	757	5		0		1		4	
Melanoma	122	3		0		1		2	
Soft tissue sarcoma	270	11		6		0		5	
Others	2635	12		2		3		7	

The values of p were based on Fisher’s exact test or Mann-Whitney tests. *In terms of age, the total number of patients was 7260 due to lack of information. **Regarding the gender, the total number of patients was 7533 due to lack of information.

### DNA Extraction, Library Construction and Sequencing

DNA was extracted from unstained FFPE sections with more than 20% tumor cells according to the manufacturer’s protocol. Library construction was performed using the KAPA Library Preparation kit. The concentration of the library was assessed using the Invitrogen Qubit4.0, and the inserted size was examined on the Agilent 4200 TapeStation. Next generation sequencing (NGS) was performed on the Illumina Novaseq 6000 system at an average depth of 1000X with a panel of 539 cancer-related genes (**Supplementary** **Table 1**). Genomic alterations, including single nucleotide variants (SNVs), copy number variations (CNVs), small insertions, deletions and gene arrangements were covered. The tumor mutation burden (TMB) and microsatellite status (MSI) were also calculated by NGS.

### Statistical Analysis

The Fisher’s exact test and Mann-Whitney test were used to assess the association of *ALK*, *ROS1*, and *NTRK* fusions with age, gender, and cancer types. To assess the probability of gene fusions in various cancer types, odds ratios (ORs) and relative 95% confidence intervals (CIs) were calculated. The overall survival (OS) was analyzed using the Kaplan-Meier method, and survival curves (mutational signature, SNVs, and CNVs) were compared using the log-rank test. Fisher’s exact test was used to evaluate the association of genomic characteristics with the proportion of PD-L1 expression, with 1% and 50% as the cutoff value. All statistical tests were two-sided, and *p* < 0.05 was considered statistically significant.

## Results

### Patient Characteristics

There were 103 (1.37%) cases harboring *ALK* rearrangements in 7,537 solid tumor patients, including lung cancer (n=93), soft tissue sarcoma (n=6), bone tumor (n=1), esophagus cancer (n=1) and other unspecified tumors (n=2). *ROS1* rearrangements were detected in 40 cases (0.53%), among whom 33 cases suffered from lung cancer (**Table 1**). 37 cases (0.49%) harbored *NTRK* fusions, including 9 cases of *NTRK1* fusions, 2 cases of *NTRK2* fusions, and 26 cases of *NTRK3* fusions.

Through NGS, a total of 180 patients were found to harbor *ALK/ROS1/NTRK* fusions and were set as *ALK/ROS1/NTRK* fusion-positive group (n=180), while those without *ALK/ROS1/NTRK* fusions were as *ALK/ROS1/NTRK* fusion-negative group (n=7357). As shown in **Table 1**, *ALK/ROS1/NTRK* fusions were more common in young patients (*p*<0.001). However, no age bias was presented in patients with *NTRK* fusion (*p*>0.05). There was a higher *ALK/ROS1/NTRK* fusion-positive frequency in females than males (*p*=0.0137), and subgroup analysis further showed that the *ROS1* fusion-positive rate in females was significantly higher than that in males (*p*=0.0006), but not *ALK* fusion (*p*=0.1906) and *NTRK* fusion (*p*=0.8671). The incidence of RTK fusions in soft tissue sarcoma and bone tumor was significantly higher than that in liver cancer (*p*<0.05). Meanwhile, the rates of RTK fusions in bile duct carcinoma and liver cancer was much lower than that in lung cancer (*p*<0.05, OR=0.285) (**Supplemental** **Figure 1**).

**Figure 1:**
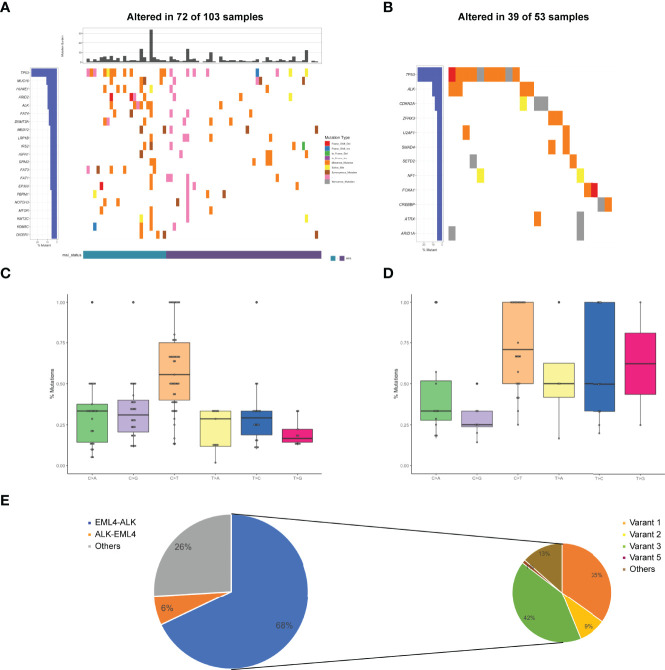
Mutational profiles and partners of *ALK* fusion-positive patients. **(A)** The oncoprint of the somatic SNVs in 103 patients harboring *ALK* fusion in our study. **(B)** The oncoprint of the somatic SNVs in 53 patients harboring *ALK* fusion in the MSKCC database. **(C)** Mutational signatures of *ALK* fusion-positive patients in our cohort. **(D)** Mutational signatures of *ALK* fusion-positive patients in the MSKCC cohort. **(E)** Distribution of *ALK* fusion partners and *EML4-ALK* variants. MSI, microsatellite instability.

### Molecular Features of *ALK* Fusion-Positive Tumors

Of 103 *ALK* fusion-positive samples, a total of 491 variants were identified, including frame InDel, missense mutations, nonsense mutations and splicing mutations. *TP53* alterations (26%) were the most common, followed by *MUC16* (11%), *HUWE1* (10%), *ARID2* (10%), and *ALK* (10%). Other genomic alterations included *NOTCH3* (6%), *MTOR* (6%), *KMT2C* (6%), *KDM5C* (6%), and *DICER1* (6%) (**Figure 1A**). The median TMB was 2.21 mut/Mb (0-33.82 mut/Mb). Although MSI status was available in 47% of patients, there was a higher proportion of microsatellite stability (MSS) in the tumors bearing *ALK* fusions. In the MSKCC cohort, totally 94 mutations occurred in 53 *ALK* fusion-positive cases, suggesting *TP53* and *ALK* were the most frequently altered genes (**Figure 1B**).

Analysis of mutational signatures showed that C>T transition were the most common, followed by C>A and C>G transitions (**Figure 1C**). The probability of T>G and T>A transitions was the lowest, consistent with COSMIC signature 1 identified in most cancer samples. Accordingly, our results were highly in accordance with MSKCC findings that C>T transition was the most frequently mutation (**Figure 1D**). Additionally, the breakpoints corresponding to *ALK* fusion in the sequencing data of these patients were also identified. Most of breakpoints were located at the intron between exon 19 and exon 20 of *ALK* gene. In the *ALK* cohort, *EML4-ALK* fusion accounted for 67%, *ALK-EML4* fusion for 6%, and others for the remaining 27%. Coexistence of these fusions was present in 26 patients (25%). 89 out of 103 patients had an *EML4-ALK* fusion, with variant 1 (v1, E13:A20), variant 2 (v2, E20:A20), variant 3 (v3, E6:A20) and variant 5 (v5, E2:A20) detected in 31, 8, 37 and 1 patients, respectively (**Figure 1E**). Sixteen novel *ALK* fusion partners identified were shown in **Supplementary** **Table 2**.

**Table 2:** Summary of PD-L1 expression in patients with *ALK*, *ROS1*, and *NTRK* fusions, n (%).

Variables		1% Cutoff			50% Cutoff		
		≥1%	<1%	*p*	≥50%	<50%	*p*
*ALK* fusion	Positive	23 (63.89)	13 (36.11)	0.0017	6 (16.67)	30 (83.33)	0.0484
	Negative	1246 (32.99)	2055 (67.01)		244 (7.39)	3057 (92.61)	
*ROS1* fusion	Positive	11 (78.57)	3(21.43)	0.0036	2 (14.29)	12 (85.71)	0.2827
	Negative	1258 (37.86)	2065 (62.14)		248 (7.46)	3075 (92.54)	
*NTRK* fusion	Positive	11 (61.11)	7 (38.89)	0.0520	1 (5.56)	17 (94.44)	1.0000
	Negative	1258 (37.90)	2061 (62.10)		249 (7.50)	3070 (92.50)	
*ALK/ROS1/NTRK* fusion	Positive	45 (66.18)	23 (33.82)	<0.0001	9 (13.24)	59 (86.76)	0.0961
	Negative	1224 (37.44)	2045 (62.56)		241 (7.37)	3028 (92.63)	

The values of p were based on Fisher’s exact test.

Notably, in our cohort, the mutations at the site of *ALK* resistance were detected. The gatekeeper L1196M (4/103) was present in crizotinib-resistant cases, while the solvent-front *G1202R* mutation (2/103) was highly resistant to crizotinib, as well as to next-generation ALK inhibitors (21).

### Molecular Features of *ROS1* Fusion-Positive Tumors

**Figure 2:**
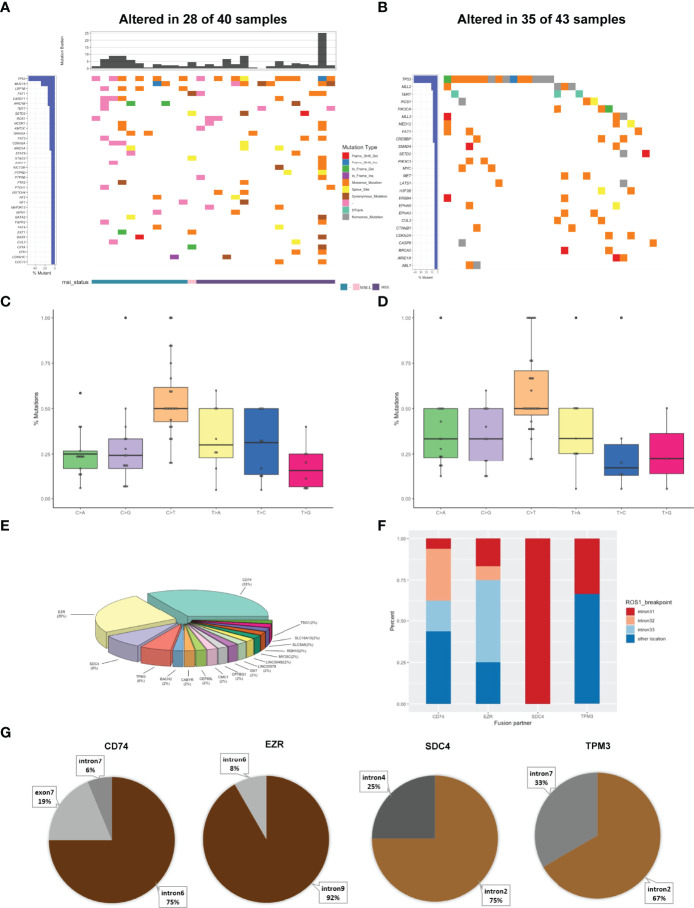
Mutational profiles and partners of *ROS1* fusion-positive patients. **(A)** The oncoprint of the somatic SNVs in 40 patients harboring *ROS1* fusion in our study. **(B)** The oncoprint of the somatic SNVs in 43 patients harboring *ROS1* fusion in the MSKCC database. **(C)** Mutational signatures of *ROS1* fusion-positive patients in our cohort. **(D)** Mutational signatures of *ROS1* fusion-positive patients in the MSKCC cohort. **(E)** Distribution of *ROS1* fusion partners. **(F)** Distribution of fusion breakpoint positions in the most common *ROS1* fusions including *CD74-ROS1*, *EZR-ROS1*, *SDC4-ROS1*, and *TPM3-ROS1*. **(G)** Distribution of breakpoint locations for *ROS1* fusion partner genes, including *CD74*, *EZR*, *SDC4*, and *TPM3*.

Genomic alterations in *ROS1* fusion-positive samples (n=40) were shown in **Figure 2A**. The median TMB was 2.94 mut/Mb with a range of 0-25 mut/Mb. 61% of *ROS1* fusion-positive tumors harboring MSI data showed MSS, while only one case showed MSI-L. The frequency of *TP53* mutations was obviously the highest (54%), followed by *MUC16* (29%), *LRP18* (14%), *FAT1* (14%), *CARD11* (14%), and *ARID18* (14%) mutations. We further compared our results with the MSKCC cohort that included 43 *ROS1* fusion-positive cases harboring 155 mutations. *TP53* was the most frequently altered gene in the MSKCC cohort, followed by *MLL2* instead of *MUC1*6 (**Figure 2B**). Analysis of their mutational signatures showed that C>T transition was the most prevalent, followed by T>A and T>C transitions (**Figure 2C**). The T>G transition showed the lowest frequency. This pattern was also consistent with COSMIC signature 1. Moreover, C>T transition also occurred most frequently in the MSKCC cohort (**Figure 2D**).

Then we identified the breakpoints and partner genes of the *ROS1* fusion in the sequencing data of these patients. In our cohort, *CD74* was the most common *ROS1* fusion partner (33%), followed by *EZR* (25%), *SDC4* (8%), *TPM3* (6%) and more (**Figure 2E**). *ROS1* fusions were formed *via* intra chromosomes, most frequently occurring in *ROS1* introns 31, 32, 33, while less frequently in other exons and introns (**Figure 2F**). Meanwhile, *ROS1* most frequently fused to intron 6 of *CD74*, intron 9 of *EZR*, intron 2 of *SDC4*, and intron 7 of *TPM3* (**Figure 2G**). We also identified 11 novel *ROS1* fusion partners (**Supplementary** **Table 2**). Mutations resulting in substitutions at solvent-front residues (G2032R) of *ROS1* were identified in one *CD74-ROS1* fusion case. The *G2032R* mutation had been reported to introduce steric hindrance and diminish high-affinity crizotinib binding (22).

### Molecular Features of *NTRK* Fusion-Positive Tumors

**Figure 3:**
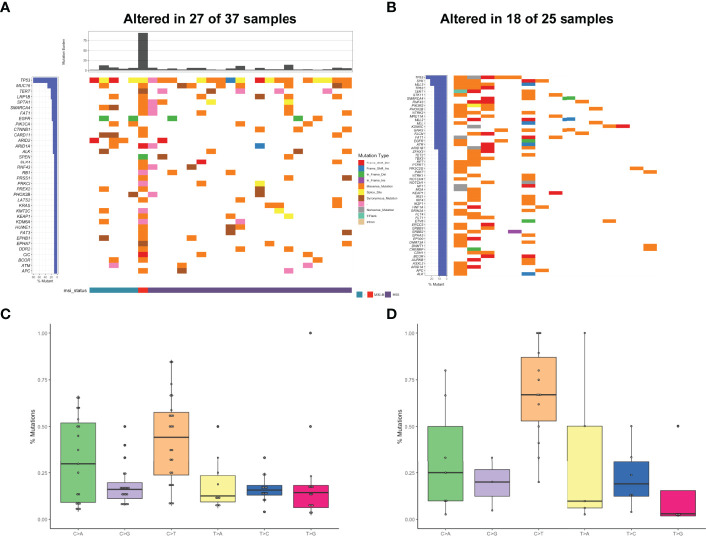
Mutational profiles and partners of *NTRK* fusion-positive patients. **(A)** The oncoprint of the somatic SNVs in 37 patients harboring *NTRK* fusion in our study. **(B)** The oncoprint of the somatic SNVs in 25 patients harboring *NTRK* fusion in the MSKCC database. **(C)** Mutational signatures of *NTRK* fusion positive patients in our cohort. **(D)** Mutational signatures of *NTRK* fusion-positive patients in the MSKCC cohort.

Among 37 *NTRK* fusion-positive cases (0.49%, 37/7537), 479 variants were totally identified in our cohort (**Figure 3A**). The median TMB was 4.41 mut/Mb, with the peak value of 93.38 mut/Mb. In the cases with MSI data, only one case bearing rearrangements was MSI-H and the others were MSS.

The heatmap of somatic mutations showed that *TP53* was the most altered gene (81%), followed by *MUC16* (33%), *TERT* (22%), *LRP1B* (22%), *SPTA1* (19%), *SMARCA4* (19%), *FAT1* (19%), and *EGFR* (19%) mutations. By analysis of the MSKCC cohort that comprised 25 *NTRK* fusion-positive cases harboring 299 mutations, *TP53* was also found to be the most frequently altered gene, followed by *SYK* but not *MUC16* (**Figure 3B**). Analysis of the mutational signatures showed that C>T transition occurred most frequently, followed by C>A transition (**Figure 3C**). The other transitions were at a low frequency. As shown in **Figure 3D**, the frequency of C>T transition in the MSKCC cohort was the highest, even higher than ours, which might be associated with different ethnicities and diets.

The positive rates of *NTRK* fusions were generally low in a wide range of cancers and tended to be enriched among rare cancers. By analyzing its partner genes, we found the proportion of *NTRK3* partner genes was the highest (76%), followed by *NTRK1* (17%), and *NTRK2* (7%). Meanwhile, 35 novel *NTRK* fusions were identified (**Supplementary** **Table 2**). Notably, *G709C* mutation resulting in amino acid substitutions was identified in the *QKI-NTRK2* fusion case, which involved the regions of the xDFG motif and was paralogous to *G1269* (*ALK*) substitutions (23).

### Classification of *ALK*, *ROS1*, and *NTRK* Fusion Events

**Figure 4:**
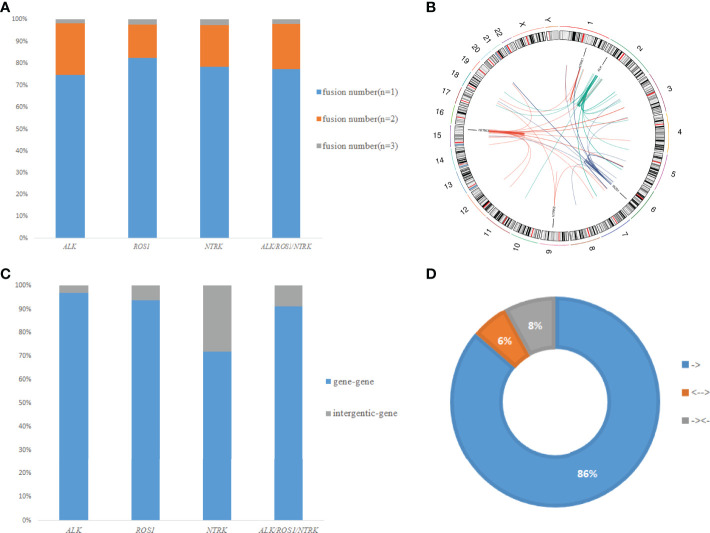
Classification of fusion events. **(A)** Distribution of different fusion numbers (n=1, 2, 3) in our study. **(B)** A circos plot of 225 gene fusions identified in all patients. **(C)** Distribution of different fusion types (gene-gene and gene-intergenic). **(D)** Distribution of fusions with different formation directions.

As shown in **Figure 4A**, a total of 225 gene fusions were identified in 180 samples, in which coexistence was present in 41 cases. These fusions occurred mostly in chromosomes, with a few occurring between adjacent chromosomes (**Figure 4B**). These fusions were classified into two categories based on the genome annotation containing the breakpoint regions (**Figure 4C**): gene-gene (91.1%) and gene-intergenic (8.9%). The gene-intergenic fusions accounted for 3.1% of *ALK* fusions, 6.3% of *ROS1* fusions, and 28.2% of *NTRK* fusions. Based on the gene breakpoint regions, we discovered 14% fusions harboring rare fusion directions, namely “upstream-upstream-breakpoint” cases (8%) and “downstream-downstream-breakpoint” cases (6%) (**Figure 4D**). Due to lack of chimeric transcripts, they were set aside in most fusion analyses as being unlikely to be functionally relevant.

### Impacts of *ALK*, *ROS1*, and *NTRK* Positivity on the Prognosis

**Figure 5:**
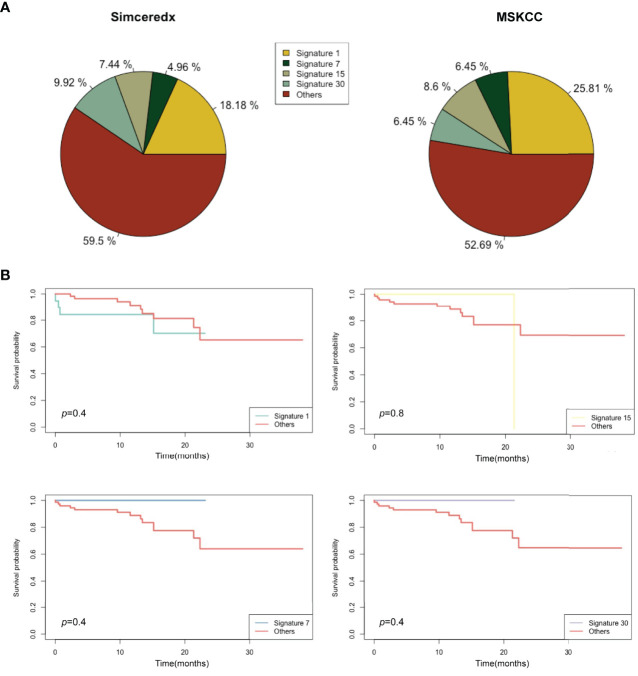
Mutational signatures of *ALK/ROS1/NTRK* fusion-positive patients. **(A)** Distribution of mutational signatures in all patients harboring *ALK/ROS1/NTRK* fusions in our study and that from the MSKCC database. **(B)** Kaplan-Meier graph for survival probability according to Sig 1, Sig 7, Sig 15, and Sig 30 status.

In this study, we used Signature Multivariate Analysis (SigMA), a computational tool, to call the mutational signatures, which could accurately detect the mutational signatures associated with homologous recombination deficiency from targeted gene panels (24). Using deconstructSigs R package to extract the mutational signatures, we identified the presence of signature 1 (Sig 1) in 18.2% (22/121), signature 3 (Sig 3) in 11.6% (14/121), signature 30 (Sig 30) in 9.9% (12/121), and signature 2 in 9% (11/121) of the patients in our cohort (**Figure 5A**). Likewise, Sig 1, signature 7 (Sig 7), signature 15 (Sig 15), and Sig 30 were detected in 25.8% (24/93), 6.5% (6/93), 8.6% (8/93), and 6.5% (6/93) of the MSKCC samples, respectively (**Figure 5B**).

A previous study indicated that Sig 3 positivity was indicative of clinical benefits (25), so we analyzed the association of Sig 1, Sig 7, Sig 15, and Sig 30 positive patients with clinical benefits. Sig 1 and Sig 15 were apparently not associated with prolonged OS (**Figure 5B**). Sig 7 and Sig 30 positive patients showed slightly longer OS than others, but without statistical significance. Interestingly, some fusion samples were absent of SNVs. On this basis, we examined whether fusion-positive samples without SNVs could indicate clinical benefits. Unfortunately, the absence of SNVs did not make a significant difference in OS (**Supplemental** **Figure 2A**).

### CNVs in Patients With *ALK*, *ROS1*, and *NTRK* Fusions

**Figure 6:**
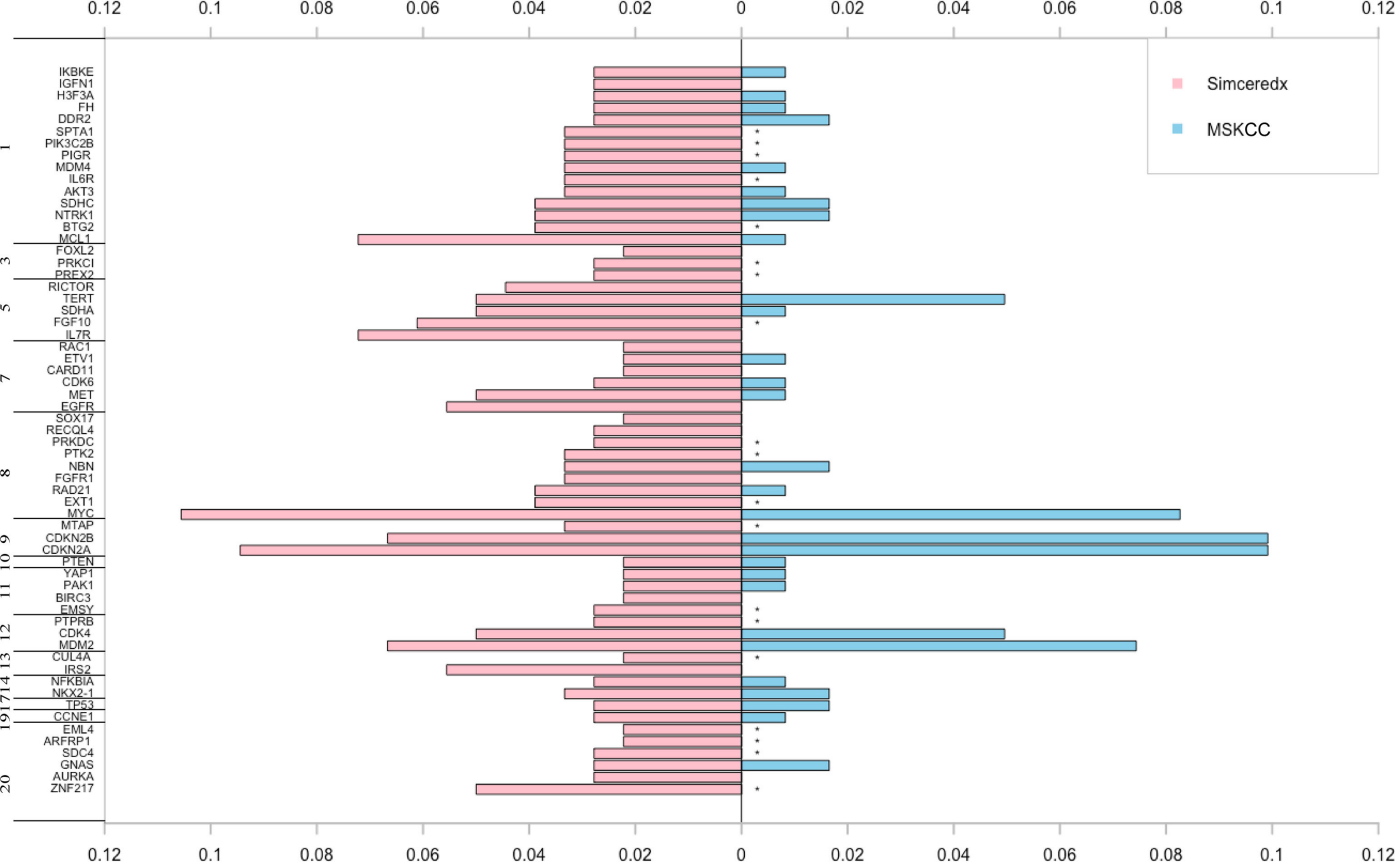
The pink and blue bars represent CNV events occurring in *ALK/ROS1/NTRK* fusion-positive patients in our cohort and MSKCC cohort, respectively. *represents the genes not covered in the MSKCC panel.

CNVs were found in 50% of 180 samples. About 11% of the patients in our cohort harbored *MYC* CNVs, which may be a candidate for tumor genesis and progression (26). In addition, CNVs of *CDKN2A*, *CDKN2B*, *MCL1*, *MDM2*, and *IRS2* have been reported to be associated with prognosis (27–31). CNVs of these genes were also found in fusion-positive samples from the MSKCC database (**Figure 6**). Interestingly, we found that CNVs of *IL7R* showed a high frequency. Moreover, the CNVs in fusion-positive samples were related to poor prognosis (*p*=0.01) (**Supplemental** **Figure 2B**), which needed more data to verify.

### PD-L1 Expression in *ALK*, *ROS1*, and *NTRK* Fusion-Positive Tumors

Over-expression of *ALK* fusion protein increased PD-L1 expression, while anti-PD-1 antibody (immunotherapy) was effective in both crizotinib sensitive and resistant NSCLC cells (32). Hence, we examined the expression of PD-L1 in our cohort. A total of 3337 patients were eligible after excluding those without PD-L1 expression. PD-L1 immunohistochemistry testing was performed using the SP263 antibody. In our cohort, PD-L1 expression was higher in tumors with *ALK* (*p*=0.0017) and *ROS1* (*p*=0.0036) fusions than fusion-negative tumors at 1% cutoff. However, PD-L1 expression between *NTRK* fusion-positive and -negative tumors showed no statistical difference (*p*=0.052). RTK fusions including *ALK*, *ROS1*, and *NTRK* exhibited a higher PD-L1 expression than fusion-negative tumors (*p*<0.0001). Using ≥50% cutoff, a higher PD-L1 positivity was also observed in tumors with *ALK* (*p*=0.0484), but not *ROS1* (*p*=0.2827), *NTRK* (*p*=1), or RTK fusions (*p*=0.0961) (**Table 2**). These results indicated that PD-L1 expression from different RTK fusion-positive tumors may have different predictive values for benefiting from immune checkpoint inhibitors (ICIs) in solid tumors.

### Aberrations in Relevant Signaling Pathways

**Figure 7:**
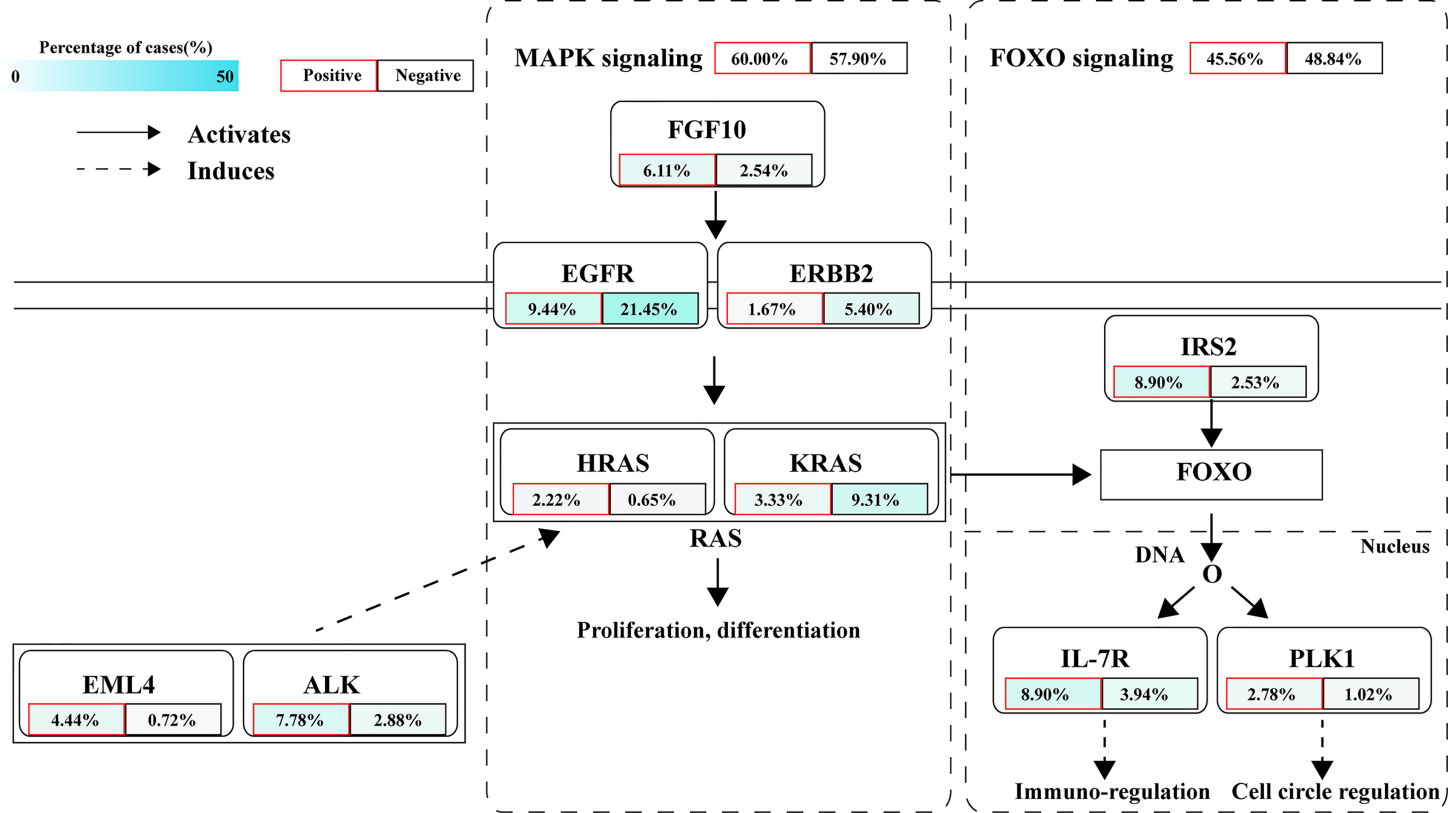
Frequently deregulated signaling pathways in *ALK/ROS1/NTRK* fusion-positive patients.

The signaling pathway analysis of *NTRK/ROS1/ALK* fusion-positive and -negative patients exhibited significant dysregulations in well-defined pathways, namely MAPK and FOXO pathways (**Figure 7**). According to prior reports, MAPK pathway regulates cell proliferation, differentiation, apoptosis, and migration, while FOXO signaling pathway is related to cell cycle, apoptosis, autophagy, metabolism, oxidation, immune response, and stem cell maintenance (33, 34). We found that the MAPK signaling pathway was altered in 60% of fusion-positive patients and 57.9% of negative patients. Fusion-positive patients had a higher frequency of mutations in *EML4*, *ALK*, *FGF10*, and *HRAS*, while the rates of *EGFR*, *ERBB2*, and *KRAS* were higher in fusion-negative patients. Differential frequencies of *IRS2*, *IL7R*, and *PLK1* mutations resulted in dysregulation of the FOXO1 signaling pathway between these two groups.

## Discussion

By analyzing the genomic landscape of patients with *ALK*, *ROS1*, and *NTRK* fusions, a relatively high frequency of *TP53* mutation, MSS status, and different TMB levels (*NTRK*>*ALK*/*ROS1*) were found, supported by previous studies (7, 9, 35, 36). In our cohort, the frequency of *MUC16* mutations was secondary to *TP53*. *MUC16* mutations appeared to be associated with the therapeutic and prognostic factors and were expected to be a biomarker to guide immunotherapy (37, 38). In terms of mutational signatures, *ALK*, *ROS1*, and *NTRK* fusion-positive patients showed similar point mutant characteristics, and the C>T transition was most common, followed by C>A transition. This pattern was consistent with COSMIC Sig 1 that had been found in most cancer types (39). Furthermore, survival curves suggested that Sig 7 and Sig 30 may be associated with a favorable prognosis in some way, which needed more data to verify.

Accumulating evidence has suggested that CNVs might be a potential biomarker or prognostic factor for tumor treatment. Apart from the genes with a high frequency of copy number amplification, such as *MYC* and *MDM2*, we identified some genes with copy number loss, such as *CDKN2A* and *CDKN2B*. *CDKN2A/B* deletions were independent prognostic markers for both adult and paediatric lymphoblastic leukaemia (40). *MDM2* amplification was associated with poor clinical outcomes and significantly increased tumor growth rates with anti-PD-1/PD-L1 immunotherapy (41). This information is important for guiding clinical treatment. We observed that fusion-positive patients without CNVs had a favorable prognosis. Notably, the pathogenic *IL-7R* CNVs exist in 7% of Chinese patients with fusions, which is higher than that in the Western population (0%). *IL7R* was previously reported to be amplified in various cancers, with the function of mediating potential tumor promotion, and high levels of *IL-7R* may be associated with poor prognosis (42). Currently, the risk factors for *IL-7R*-mutant fusions are unknown. Future studies should focus on how diet and ethnic differences increase the risk of *IL-7R* mutations.

Of the 180 fusion-positive samples by NGS, *EML4* and *CD74* were the most common *ALK* and *ROS1* fusion partners, respectively. *EML4-ALK* occurred mainly in the forms of three variants: variant 1, variant 2, and variant 3 (43, 44). Diverse *ROS1* fusion partners were identified, and the top four fusion partners were *CD74*, *EZR*, *SDC4*, and *TPM3*. As the most common ROS1 fusion partner, CD74 had a frequency similar to the previous ones (9, 45). There were no high-frequency partner genes occurring in *NTRK* fusions, which might be related to the high incidence of *NTRK* fusions in rare tumors. We also detected some novel *ALK/ROS1/NTRK* fusion partners, such as *LPIN1* and *SMARCC1* (*ALK*), *SLC16A10* and *CRYBG1* (*ROS1*)*, SDK1* and *GYPA* (*NTRK3*). These results suggested that the NGS-based evaluation for *ALK/ROS1/NTRK* fusions was accurate and comprehensive. Compared with traditional methods, such as IHC, FISH, and Sanger sequencing, NGS had unique advantages in detecting unknown fusion partners and identifying accurate breakpoints. However, the rare fusions remain clinically interesting, further studies are needed to confirm these observations in preclinical and clinical studies.

A previous study reported the impacts of gene-intergenic and intergenic-intergenic fusions on the upregulation of their target genes (46). Therefore, we classified the fusions in our cohort into two categories: gene-gene fusion and gene-intergenic fusion. Neither intergenic sequence-*ALK* nor coexistence of fusions showed a significant effect on the benefit from crizotinib treatment (47). However, a substantial portion of chimeric transcripts was produced by gene-intergenic fusions. The impact of such intergenic breakpoints on transcriptome has been unclear. Meanwhile, these fusions with rare fusion directions mostly coexisted with classic fusions, and their clinical significance was currently unknown, even though a portion of them harboring kinase domains. Future research may focus on investigating the clinical role of gene-intergenic fusions and fusions with rare fusion directions in cancers.

PD-L1 protein expression in tumor cells emerged as the first potential predictive biomarker for sensitivity to ICIs (48). In our cohort, 44.27% of the patients had clinically relevant information in PD-L1 expression. Consistent with the literature, we observed a significantly higher expression of PD-L1 in the *ALK* fusion-positive cohort. Of note, the expression of PD-L1 in the *ROS1* or *NTRK* fusion-positive cohort was similar to that in the fusion-negative cohort. However, other data suggested that immune escape may confer a higher PD-L1 expression in NSCLC patients with an aggressive tumor phenotype, leading to a poor prognosis with TKI therapy (40). Moreover, the differentially mutated genes between fusion-positive and fusion-negative samples were mainly enriched in the MAPK and FOXO signaling pathways. The mutational frequency of individual gene varied greatly between fusion-positive and fusion-negative samples, but with similar mutational frequency in the whole signaling pathways.

In conclusion, we characterized the genomic landscape of solid tumor patients with *ALK*, *ROS1*, and *NTRK* fusions and 62 novel fusions were discovered, which may provide more clinically actionable targets for cancer therapy to a great extent. Although the gene-intergenic fusion and fusion with rare fusion directions accounted for a certain proportion of all fusion samples, the clinical significance of these fusions remained to be unclear, thus RTK-targeted therapy should be explored further in solid tumors in the future. Notably, the frequency of CNVs was high and associated with a poor prognosis in fusion-positive patients, highlighting the importance of CNVs as a potential biomarker or prognostic factor for cancer therapy. PD-L1 high-expression was more common in the *ALK* fusion-positive cohort than that in the fusion-negative cohort, leading us to hypothesize that ICIs might bring clinical benefits to the solid tumor patients harboring RTK fusions. Collectively, all these findings may provide genomic information for personalized clinical management of patients with *ALK*, *ROS1*, and *NTRK* fusions in the era of precision medicine.

## Data Availability Statement

The datasets presented in this study can be found in online repositories. The names of the repository/repositories and accession number(s) can be found below: GenBank, submission #2562890.

## Ethics Statement

Ethical review and approval was not required for the study on human participants in accordance with the local legislation and institutional requirements. The patients/participants provided their written informed consent to participate in this study.

## Author Contributions

YD, CS, YL and YS contributed to conception and design of the study. YD wrote the first draft of the manuscript. PL, WH, LY wrote sections of the manuscript. YN, XM and FD organized the database and performed the statistical analysis. CS, YL and YS contributed to edit and review the manuscript. All authors approved the submitted version.

## Funding

This study was supported by Hunan Principal Project “The molecular mechanism of the reversion of glioma differentiation induced by Toll-like receptor 4 activation of Notch signal pathway” (ID: 2019JJ40422).

## Conflict of Interest

Authors YN, XM, FD, and CS were employed by Jiangsu Simcere Diagnostics Co., Ltd. And Nanjing Simcere Medical Laboratory Science Co., Ltd.

The remaining authors declare that the research was conducted in the absence of any commercial or financial relationships that could be construed as a potential conflict of interest.

## Publisher’s Note

All claims expressed in this article are solely those of the authors and do not necessarily represent those of their affiliated organizations, or those of the publisher, the editors and the reviewers. Any product that may be evaluated in this article, or claim that may be made by its manufacturer, is not guaranteed or endorsed by the publisher.
