# Supplementary material for: Genomic Features of Solid Tumor Patients Harboring ALK/ROS1/NTRK Gene Fusions
Source: Front Oncol. 2022 Jun 16;12:813158. doi: 10.3389/fonc.2022.813158 (PMC9243239; doi:10.3389/fonc.2022.813158)
Supplement: Supplementary file 1 [file DataSheet_1.docx]

Supplementary Material

**Supplementary Table 1. The list of genes included in a 539 cancer-targeted gene panel**

| A2M | ABCB1 | ABL1 | ACTL6A | ACTL6B | ACVR1B | ADH1B | AKT1 | AKT2 | AKT3 |
| --- | --- | --- | --- | --- | --- | --- | --- | --- | --- |
| ALDH2 | ALK | ALOX12B | AMER1 | APC | APLNR | AR | ARAF | ARFRP1 | ARID1A |
| ARID1B | ARID2 | ARID5B | ASXL1 | ATG13 | ATG2A | ATG7 | ATM | ATR | ATRX |
| AURKA | AURKB | AXIN1 | AXIN2 | AXL | B2M | BAK1 | BAP1 | BARD1 | BCL10 |
| BCL2 | BCL2L1 | BCL2L11 | BCL2L2 | BCL6 | BCOR | BCORL1 | BCR | BIRC3 | BLM |
| BMPR1A | BRAF | BRCA1 | BRCA2 | BRD4 | BRD7 | BRIP1 | BTG1 | BTG2 | BTK |
| BUB1B | C10orf11 | C11orf30 | CALR | CARD11 | CASP8 | CBFB | CBL | CBR3 | CCND1 |
| CCND2 | CCND3 | CCNE1 | CD274 | CD70 | CD74 | CD79A | CD79B | CDA | CDC42 |
| CDC73 | CDH1 | CDK12 | CDK4 | CDK6 | CDK8 | CDKN1A | CDKN1B | CDKN1C | CDKN2A |
| CDKN2B | CDKN2C | CEBPA | CFH | CFHR1 | CHD4 | CHEK1 | CHEK2 | CIC | CIITA |
| CREBBP | CRKL | CRLF2 | CSF1R | CSF3R | CTCF | CTLA4 | CTNNA1 | CTNNB1 | CUL3 |
| CUL4A | CXCR4 | CYLD | CYP17A1 | CYP19A1 | CYP2C19 | CYP2C8 | CYP2D6 | CYP2E1 | DAPK1 |
| DAXX | DDR1 | DDR2 | DHFR | DICER1 | DIS3 | DNMT1 | DNMT3A | DOT1L | DPYD |
| DYNC2H1 | EED | EGFR | EML4 | EP300 | EPAS1 | EPCAM | EPHA2 | EPHA3 | EPHA5 |
| EPHA7 | EPHB1 | EPHB4 | ERBB2 | ERBB3 | ERBB4 | ERCC1 | ERCC2 | ERCC3 | ERCC4 |
| ERCC5 | ERG | ERRFI1 | ESR1 | ETV1 | ETV4 | ETV5 | ETV6 | EWSR1 | EXT1 |
| EXT2 | EZH2 | EZR | FAM175A | FAM46C | FANCA | FANCC | FANCD2 | FANCE | FANCF |
| FANCG | FANCI | FANCL | FAS | FAT1 | FAT2 | FAT3 | FAT4 | FBXW7 | FGA |
| FGF10 | FGF12 | FGF14 | FGF19 | FGF23 | FGF3 | FGF4 | FGF6 | FGFR1 | FGFR2 |
| FGFR3 | FGFR4 | FH | FLCN | FLT1 | FLT3 | FLT4 | FOXA1 | FOXL2 | FOXO1 |
| FOXP1 | FUBP1 | FYN | FZR1 | GABRA6 | GATA1 | GATA2 | GATA3 | GATA4 | GATA6 |
| GGH | GLI1 | GNA11 | GNA13 | GNAQ | GNAS | GREM1 | GRIN2A | GRM3 | GSK3B |
| GSTM1 | GSTP1 | GSTT1 | H3F3A | H3F3B | H3F3C | HDAC1 | HDAC2 | HGF | HIST1H1C |
| HIST1H2BD | HIST1H3A | HIST1H3B | HIST1H3C | HIST1H3D | HIST1H3E | HIST1H3G | HIST1H3H | HIST1H3I | HIST1H3J |
| HIST2H3D | HIST3H3 | HLA-A | HLA-B | HLA-C | HLA-DQB1 | HNF1A | HNF1B | HRAS | HSD3B1 |
| HUWE1 | ID3 | IDH1 | IDH2 | IFNGR1 | IFNGR2 | IGF1 | IGF1R | IGF2 | IGHN1 |
| IKBKE | IKZF1 | IL6R | IL6ST | IL7R | INHBA | INPP4B | INSR | IRF2 | IRF4 |
| IRS1 | IRS2 | JAK1 | JAK2 | JAK3 | JUN | KDM5A | KDM5C | KDM6A | KDR |
| KEAP1 | KEL | KIT | KLF4 | KLHL6 | KMT2A | KMT2C | KRAS | LATS1 | LATS2 |
| LIG3 | LMO1 | LRP1B | LTK | LYN | MAF | MAP2K1 | MAP2K2 | MAP2K4 | MAP3K1 |
| MAP3K13 | MAPK1 | MAPK3 | MAX | MCL1 | MDM2 | MDM4 | MED12 | MEF2B | MEN1 |
| MERTK | MET | MITF | MKNK1 | MLH1 | MPL | MRE11A | MSH2 | MSH3 | MSH6 |
| MST1R | MTAP | MTHFR | MTOR | MUC16 | MUTYH | MYB | MYC | MYCL | MYCN |
| MYD88 | NBN | NCOR1 | NF1 | NF2 | NFE2L1 | NFE2L2 | NFKBIA | NKX2-1 | NKX3-1 |
| NOTCH1 | NOTCH2 | NOTCH3 | NOTCH4 | NPM1 | NQO1 | NRAS | NSD1 | NT5C2 | NTHL1 |
| NTRK1/2/3 | NUP93 | NUTM1 | P2RY8 | PAK1 | PAK3 | PAK7 | PALB2 | PARK2 | PARP1 |
| PARP2 | PARP3 | PAX5 | PBRM1 | PDCD1 | PDCD1LG2 | PDE4D | PDGFRA | PDGFRB | PDK1 |
| PHOX2B | PIGR | PIK3C2B | PIK3C2G | PIK3C3 | PIK3CA | PIK3CB | PIK3CG | PIK3R1 | PIK3R2 |
| PIM1 | PLCG2 | PLK1 | PMS1 | PMS2 | POLD1 | POLE | PPARG | PPP2R1A | PRDM1 |
| PREX2 | PRKAR1A | PRKCI | PRKDC | PRSS1 | PTCH1 | PTEN | PTK2 | PTPN11 | PTPRB |
| PTPRD | PTPRO | QKI | RAC1 | RAC2 | RAD17 | RAD21 | RAD50 | RAD51 | RAD51B |
| RAD51C | RAD51D | RAD52 | RAD54L | RAF1 | RARA | RB1 | RBM10 | RECQL4 | REL |
| RET | RHEB | RHOA | RICTOR | RNF43 | ROS1 | RPS6KA3 | RPTOR | RRM1 | RSPO2 |
| RUNX1 | RXRA | SBDS | SDC4 | SDHA | SDHAF2 | SDHB | SDHC | SDHD | SERPINB3 |
| SERPINB4 | SETD2 | SF3B1 | SGK1 | SH2D1A | SLC34A2 | SLCO1B1 | SXL4 | SMAD2 | SMAD3 |
| SMAD4 | SMARCA1 | SMARCA2 | SMARCA4 | SMARCB1 | SMARCC1 | SMARCC2 | SMARCD1 | SMARCE1 | SMO |
| SNCAIP | SOCS1 | SOD2 | SOS1 | SOX17 | SOX2 | SOX9 | SPEN | SPOP | SPTA1 |
| SRC | SRSF2 | STAG2 | STAT1 | STAT2 | STAT3 | STAT4 | STAT5A | STAT5B | STAT6 |
| STK11 | SUFU | SUZ12 | SYK | TBX3 | TCF7L2 | TEK | TERT | TET1 | TET2 |
| TFG | TGFBR1 | TGFBR2 | THADA | TIPARP | TMEM127 | TMEM173 | TMPRSS2 | TNFAIP3 | TNFRSF11A |
| TNFRSF14 | TNFSF11 | TOP1 | TOP2A | TP53 | TP63 | TPMT | TRAF7 | TSC1 | TSC2 |
| TSHR | TYMS | TYRO3 | U2AF1 | UGT1A1 | UMPS | VEGFA | VHL | VTCN1 | WAS |
| WHSC1 | WHSC1L1 | WISP3 | WNT10A | WNT10B | WNT7B | WRN | WT1 | XIAP | XIAP |
| XPA | XPC | XPO1 | XRCC1 | XRCC2 | YAP1 | YES1 | ZNF217 | ZNF703 |  |

**Supplementary Table 2.** Novel Partners of *ALK*, *ROS1*, and *NTRK* rearrangements

| Partner-*ALK* (n=2) | *ALK*-Partner (n=14) | Partner-*ROS1* (n=5) | *ROS1*-Partner (n=6) | Partner-*NTRK* (n=22) | *NTRK*-Partner (n=13) | Frequency |
| --- | --- | --- | --- | --- | --- | --- |
|  |  |  |  | AGBL1 |  | 3 |
|  |  |  |  | MRPS11, RBM27, TFG |  | 2 |
| LPIN1, SMARCC1 | LLC, C2orf71, CEP350, CTNNA2, DNAJC27, GOLGA4, HAAO, LINC01194, LMLN, LOC102723824, MAP4K3, SFTPB, SLC38A10, WDR43 | CABYR, CRYBG1, DST, LINC00578, SLC16A10 | BACH2, CMC1, LINC00492, RDH10, SLC5A9,  TSG1 | ALPK3, DAMTS17, FAM227B, GYPA, KLHL6, LINGO1, LOC440311, PIAS2, SDK1, SYPL1, TMEM200C, TMTC2，  XRCC3 | ABHD2, ADAMTSL3, BAHD1, BLM, CHD2, C15orf38-AP3S2, IQGAP3, KLHL24, LINC01815, MTHFS, NCOA6, SLC28A3, UACA | 1 |

## Supplementary Figures


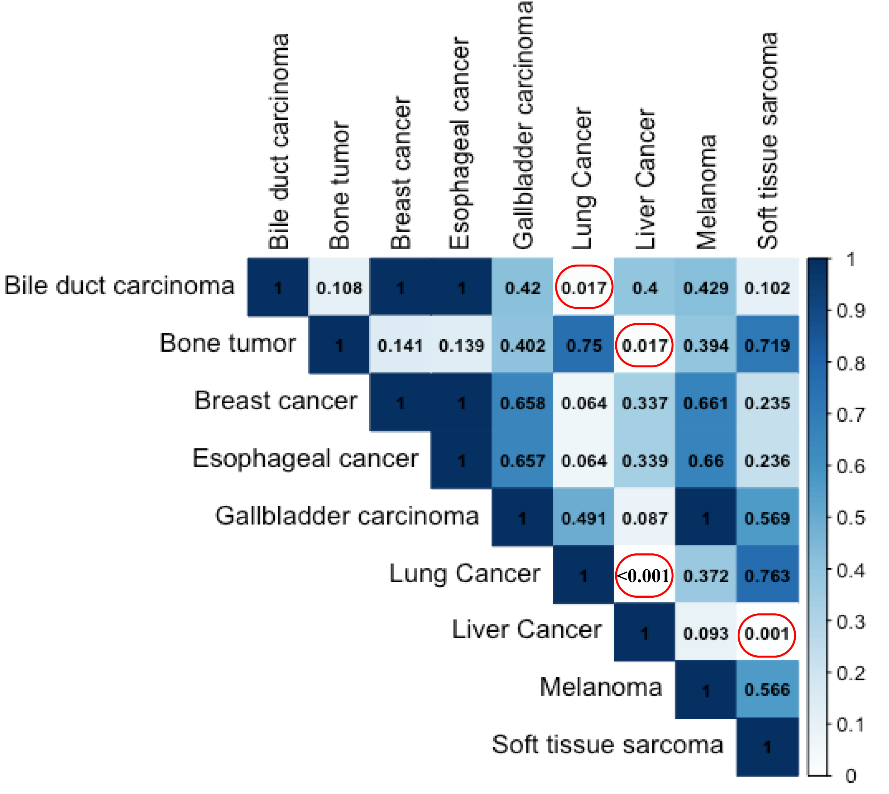


**Supplementary Figure 1**


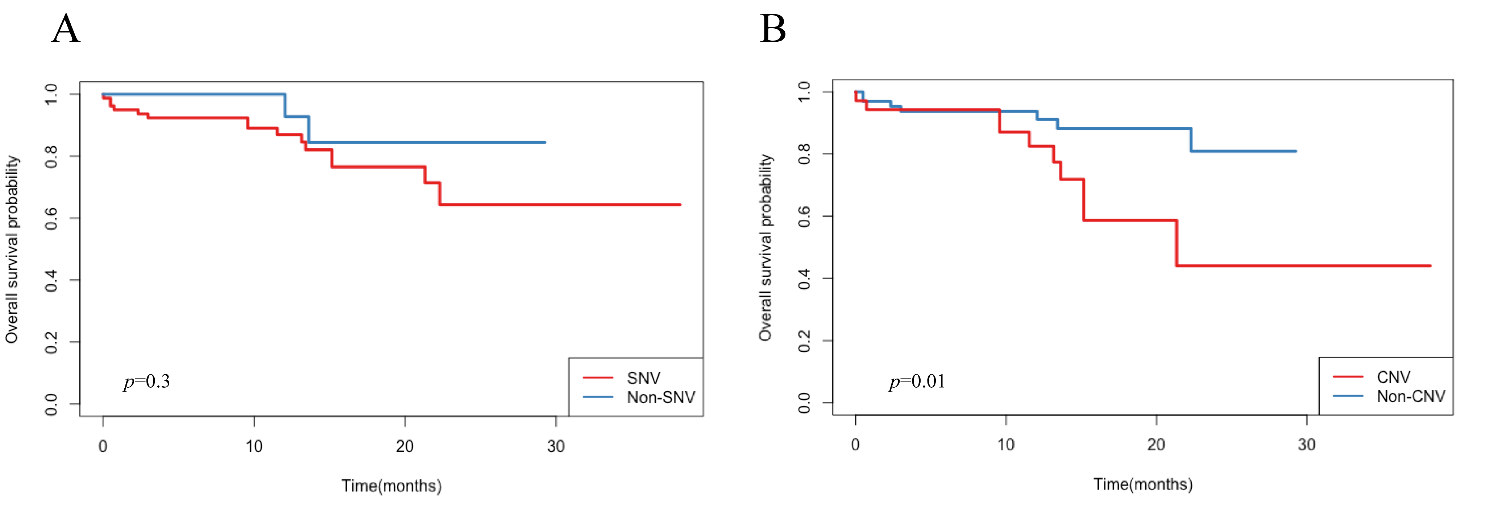
**Supplementary Figure 2**

**Supplemental Figure 1** Comparison on the distribution of *ALK/ROS1/NTRK* fusions in different cancers. Significant differences were observed between bile duct carinoma and lung cancer, bone tumor and liver cancer, soft tissue sarcoma and liver cancer, lung cancer and liver cancer.

**Supplemental Figure 2** Overall survival in *ALK*/*ROS1*/*NTRK* fusion-positive patients carrying SNVs and CNVs. (A) Kaplan-Meier curves for OS in patients with RTK fusions and SNVs as compared with those with RTK fusions and non-SNV tumors. (B) Kaplan-Meier curves for OS in patients with RTK fusion and CNVs as compared with those with RTK fusion and non-CNV tumors.
